# The Impacts of Animal-Based Diets in Cardiovascular Disease Development: A Cellular and Physiological Overview

**DOI:** 10.3390/jcdd10070282

**Published:** 2023-06-30

**Authors:** Rami Salim Najjar

**Affiliations:** Institute for Biomedical Sciences, Georgia State University, Atlanta, GA 30303, USA; rnajjar1@gsu.edu

**Keywords:** plant-based diet, low-carbohydrate diet, TLR4, oxidized LDL, lipotoxicity, endothelial function, atherosclerosis, hypertension, heart failure, polyphenols, saturated fat

## Abstract

Cardiovascular disease (CVD) is the leading cause of death in the United States, and diet plays an instrumental role in CVD development. Plant-based diets have been strongly tied to a reduction in CVD incidence. In contrast, animal food consumption may increase CVD risk. While increased serum low-density lipoprotein (LDL) cholesterol concentrations are an established risk factor which may partially explain the positive association with animal foods and CVD, numerous other biochemical factors are also at play. Thus, the aim of this review is to summarize the major cellular and molecular effects of animal food consumption in relation to CVD development. Animal-food-centered diets may (1) increase cardiovascular toll-like receptor (TLR) signaling, due to increased serum endotoxins and oxidized LDL cholesterol, (2) increase cardiovascular lipotoxicity, (3) increase renin-angiotensin system components and subsequent angiotensin II type-1 receptor (AT1R) signaling and (4) increase serum trimethylamine-N-oxide concentrations. These nutritionally mediated factors independently increase cardiovascular oxidative stress and inflammation and are all independently tied to CVD development. Public policy efforts should continue to advocate for the consumption of a mostly plant-based diet, with the minimization of animal-based foods.

## 1. Introduction

A variety of dietary approaches have emerged in recent years aimed at addressing the chronic disease epidemic [1], including cardiovascular disease (CVD). However, the spectrum of these diets varies extensively with respect to their composition. For example, diets such as the ketogenic diet advocate for the near elimination of carbohydrates while simultaneously encouraging the consumption of copious amounts of fat and moderate intake of protein [2]. Other low carbohydrate diet models replicate aspects of this with changes in protein or fat proportionally, such as the paleolithic diet, the Atkins diet or the “carnivore” diet. In contrast, a whole-food, plant-based diet tends to be higher in carbohydrates and lower in fat with moderate-to-lower protein intake [3]. Macronutrients, however are not consumed in isolation of the food from which they are derived; diets which favor high protein or fat consumption tend to also be animal-based, with far fewer (or near-absent) calories, derived from plant foods. While humans are an adaptable species nutritionally, our nutritional biology is not wildly different. This is evident in the study of the “Blue Zones”, which illustrate common dietary themes among the most long-lived populations from different regions of the world who have very low CVD incidence [4]. Individuals in these groups tend to eat diets higher in plant foods derived from unprocessed carbohydrates and also consume fewer animal products. These include the Okinawans from Japan, Seventh-Day Adventists from Loma Linda, CA, USA, the Nicoyans from Costa Rica, the Sardinians from Italy and the Ikarians from Greece.

Illustrative of the Blue Zone diets, the Okinawans in the 20th century had the highest number of centenarians per capita [5]. Their diet was 85% percent carbohydrates derived from whole plant foods, such as sweet potatoes (69% of total calories), grains (19% of total calories) and legumes (6% of total calories), while animal foods comprised 1–2% of total calories of their diet. In 1995, the Okinawan CVD-associated mortality in males was nearly one-sixth the rate of male counterparts in the United States, and among Okinawan woman, CVD-associated mortality was less than one-twelfth the rate of female counterparts in the United States. Similarly, the Seventh-Day Adventists in the United States consume a mostly plant-based diet, and vegetarian men and woman have life expectancies that are 9.5 and 6.1 years greater than their Californian counterparts, respectively [6]. It appears that animal food intake is significantly tied to CVD mortality among males. Compared to omnivores, ischemic heart disease and overall CVD mortality was associated with a 24% and 23% reduction in vegetarian Adventists (dairy and eggs, but no meats), respectively, while vegan males (no animal products) had a 55% and 42% reduction in ischemic heart disease and overall CVD mortality, respectively [7]. It should be noted that the CVD-protective effects of a plant-based diet are less clear with females in this cohort.

It is recognized that genetic factors are not primary drivers of the extended lifespans observed, as Westernization, characterized by the consumption of fewer plants and more meat, has resulted in a stark increase in CVD incidence among Japanese with traditionally low CVD incidence [8]. Migration studies also indicate that integration into Western societies among Okinawan and Japanese individuals results in a shift towards the increased prevalence of CVD risk factors, which were once nearly absent from these cultures [9,10]. In contrast to the Blue Zones, the Arctic Inuit consume significantly more meat, particularly seafood, compared to their Western counterparts, and their diet is, by definition, lower in carbohydrate content [11]. However, the lifespan of Inuit is 10 years less on average than their Western counterparts, with twice the mortality, due to a high incidence of stroke [12]. While diet cannot be considered the sole factor contributing to the reduction in lifespan in this population, it is certainly a major factor considering the primary role of lifestyle in mediating stroke risk [13,14].

Animal-based diets tend to be much lower in fiber, and indeed, the consumption of fiber, which is inexorably linked to unprocessed plant-food consumption, is associated with reduced CVD incidence in the most rigorous of systematic analyses [15]. Additionally, carbohydrate consumption observed at the low end of the spectrum (~≤20% of calories) is associated with increased mortality, while carbohydrates at the high end of the spectrum (~≥70% of calories) is also associated with increased mortality [16]. However, an important caveat must be noted, in that this association observed with high-carbohydrate consumption is not evident in those consuming unprocessed carbohydrates (e.g., whole grains rather than refined grains). Indeed, the evidence which supports whole grain consumption (a rich source of carbohydrates) is compelling [17,18,19], as not a single study can be identified in the literature which demonstrates that whole grain consumption is associated with increased mortality or biomarkers classically associated with CVD. The same is true of the consumption of fruit, which collectively contain even greater percentages of calories derived from carbohydrates than grains [20]. Overall, plant-based dietary patterns characterized by the consumption of whole grains, fruits and vegetables are associated with reduced CVD incidence [21].

In addition to fiber, a major nutritional aspect unique to plants is their polyphenol content, secondary metabolites found strictly in plants which have bioactive properties. My prior work with colleagues (Feresin, Turner and Wong [22,23,24]) defined a number of molecular pathways involved in CVDs which polyphenols could beneficially target. In limited clinical investigations, my prior work with colleagues (Montgomery and Moore [25,26,27]) demonstrated that a plant-based diet could reduce blood pressure more efficaciously than hypertension-managing drugs [25], reduce atherogenic lipoproteins and inflammatory markers with high efficacy [26], and, based on data from a case series of three patients [27], potentially treat heart failure adjunctly. Jenkins et al. demonstrated that a weight-maintaining plant-based diet very high in raw fruits, vegetables and nuts could reduce low-density lipoproteins (LDL) by ~33% [28], a reduction comparably as effective as statin treatment [29]. Other investigators have shown that a plant-based diet could treat atherosclerosis, an outcome previously thought improbable through dietary means [30,31]. Overall, plant-based diets are associated with reduced blood pressure [32] and serum cholesterol concentrations [33], risk factors associated with reduced CVD.

### Animal Food-Based Diets: Are They Health Promoting?

Despite compelling population studies, epidemiological data and promising clinical data regarding the efficacy of consuming more plants for cardiovascular health, meat-based and classically high-protein/high-fat diets, such as the Atkins, paleolithic or ketogenic diet, have gained popularity in American culture. In practice, the restriction of carbohydrates will concomitantly reduce the proportion of calories derived from plants, since carbohydrates are exclusively found in plant-based foods (with the exception of small amounts found in dairy) [16]. Thus, a low-carbohydrate diet is almost always a high-animal-product diet. Interestingly however, low-carbohydrate diets which are more plant-based tend to be associated with reduced CVD mortality (21% reduction in risk) compared to more animal-based (22% increased risk) [34], illustrating that it is indeed animal-based foods which are the problem. In fact, a fully vegan, low-carbohydrate, low-saturated fat diet (26% carbohydrates, 31% protein, and 43% fat) rich in soy, wheat gluten, nuts and oils was more effective than a higher carbohydrate lacto-ovo-vegetarian diet in reducing LDL cholesterol and triglycerides after four weeks with energy restriction [35] and 6 months ad libitum [36]. In contrast, low-carbohydrate animal-based diets inherently lead to increased saturated fatty acid consumption, which results in a predictable rise in serum LDL cholesterol concentrations [37]. The American Heart Association (AHA) presidential panel position statement on the link between serum LDL cholesterol and cardiovascular events identified this link as causal [37]. Further, the consumption of animal proteins, but not plant proteins, is associated with increased CVD events [16]. Indeed, 2021 dietary guidance from AHA ranked both the paleolithic diet and very low carbohydrate diets (e.g., the ketogenic diet and the Atkins diet) as the worst diets for cardiometabolic health, and emphasized the consumption of plant foods with fewer animal foods to prevent CVDs [38].

It is interesting to note that a number of clinical trials which have utilized animal-product-rich diets (low-carbohydrate, high-protein or high-fat diets) in comparison to more mixed diets that are higher in carbohydrate and lower in fat demonstrate benefit in CVD risk factors, despite inherently reduced plant food consumption and increased saturated fat intake [39]. While this may seem paradoxical based on the aforementioned literature, no paradox truly exists. It is important to note the following two dominant methodological features which drive the appearance of benefit with poor societal extrapolation: (1) the low-carbohydrate diets are not intended to meet energy needs, and are instead designed to substantially reduce caloric consumption, leading to inherently more favorable lipid and metabolic profiles as well as blood pressure, due to reduced body weight, although LDL may be increased in some cases despite body weight reductions due to increased saturated fat intake [40], and (2) the low-fat, higher-carbohydrate dietary group which acts as the control comparison tends to be of low dietary quality, as evidenced by very low fiber intake (~15 g/day), a clear indication of processed food consumption, and is thus not designed to be health promoting. This flawed comparison group design neglects the important concept that macronutrients are not independent of the foods from which they are derived.

For example, sugary cereal, such as frosted flakes and white bread are 93% and 76% carbohydrates, respectively, compared to black beans, sweet potatoes and oats, which are 73%, 93% and 74% carbohydrates, respectively [41]. Even for lay individuals, one would consider the nutritional quality of these latter foods to be far superior compared to the former, despite similar carbohydrate contents. Illustrative of this concept, it has been demonstrated that unprocessed, plant-based diets, characterized by increased fruits, vegetables, nuts, legumes and whole grains are associated with reduced CVD mortality, but not plant-based diets with more processed foods such as refined grains, fruit juices, potatoes (French fries, potato chips, mashed potatoes) and desserts (cakes, candy, pastries) [42]. Thus, one cannot determine the nutritional quality of a moderate-to-high carbohydrate diet without knowing what foods the diet is comprised of. Fiber intake is typically a good indicator of diet quality, since it is exclusive to minimally processed plant foods.

**Figure 1:**
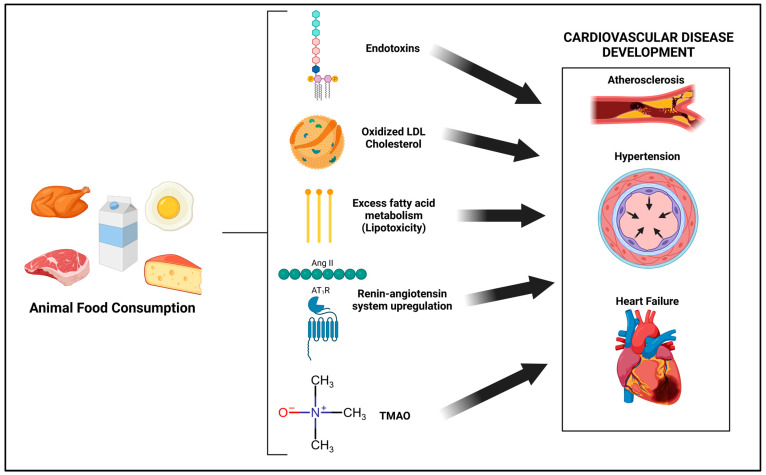
Overall mechanisms by which animal-based diets can contribute to the development of cardiovascular diseases.

To conclude that animal-product-rich diets are healthy or efficacious in treating CVD would be erroneous based on the aforementioned literature. Additionally, animal-product-rich diets tend to impair vascular function [43,44], increase LDL cholesterol and inflammation compared to an isocaloric unprocessed high-carbohydrate diet [45], and reduce myocardial blood flow compared to a plant-based diet, which had the opposite effect [46]. While increased saturated fat consumption and subsequently increased serum LDL cholesterol are a well-known and plausible physiological mechanism by which these diets are associated with CVD risk, there is far greater complexity and a multitude of other biochemical mechanisms involved that exacerbate or are independent of these effects. To date, these mechanisms have not been well-defined or discussed. Thus, the aim of this review is to examine major biochemical and molecular mechanisms by which the consumption of animal products could promote CVD risk. These overall mechanisms are highlighted in Figure 1.

## 2. Diet-Mediated Toll-Like Receptor (TLR) Activation

**Figure 2:**
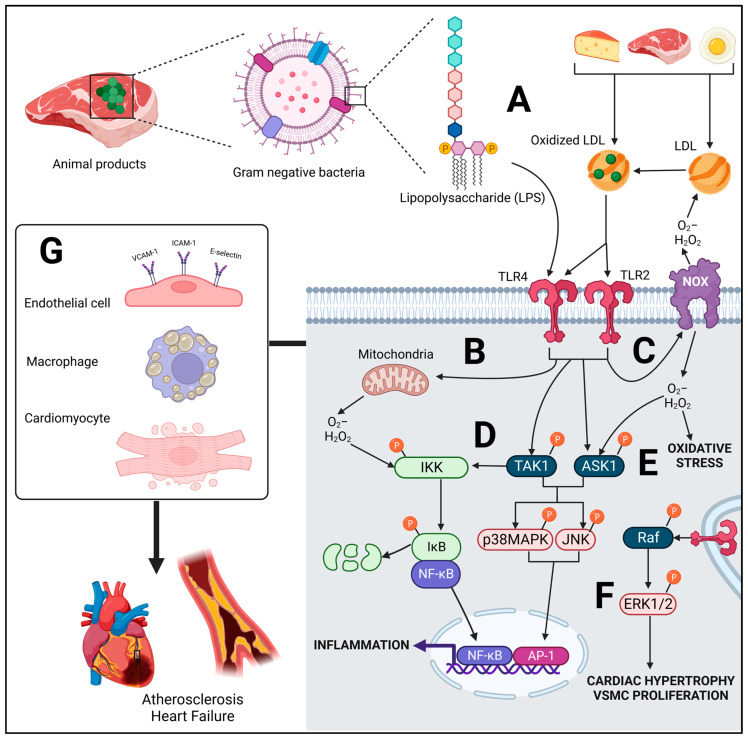
Molecular mechanisms by which TLR ligands derived from the consumption of animal-based foods can promote cardiovascular disease. (**A**) Lipopolysaccharides (LPS) and oxidized low-density lipoprotein (LDL) derived from animal-based foods can act as TLR ligands. Reactive oxygen species (ROS) derived from (**B**) mitochondria and (**C**) NADPH-oxidases (NOX) due to downstream TLR-mediated signaling can oxidize LDL directly or indirectly with secondary oxidative products, such as peroxynitrite (ONOO-). (**D**) TLR signaling can lead to the downstream phosphorylation of transforming growth factor-β-activated kinase (TAK)1, a mitogen-activated protein kinase kinase kinase (MAPKKK), which phosphorylates IκB kinase (IKK). Alternatively, ROS can induce autophosphorylation of IKK due to reactive cysteine residues. IKK signaling induces phosphorylation of IκB, which then phosphorylates nuclear factor-κB (NF-κB), a nuclear transcription factor that translocates to the nucleus and undergoes DNA binding to induce pro-inflammatory gene expression. (**E**) The MAPKKK, apoptosis signal-regulating kinase 1 (ASK1) is also activated by TLRs or ROS to phosphorylate p38MAPK and c-Jun N-terminal kinase (JNK), with TAK1 having similar effects. Phosphorylation of these terminal MAPKs results in the nuclear translocation of the activator protein 1 (AP-1) transcription factor family to also induce pro-inflammatory gene expression. (**F**) The MAPKKK, Raf, is activated by TLR4 and its activation leads to the phosphorylation of ERK1/2 involved in mediating cardiac hypertrophy and the proliferation of vascular smooth muscle cells (VSMCs). (**G**) These molecular effects at the cellular level drive the development of CVDs, such as atherosclerosis and heart failure. These include endothelial dysfunction and the expression of leukocyte adhesion molecules, the activation of macrophages and foam cell formation in the subendothelial space, as well as cardiomyocyte hypertrophy and eventually apoptosis, as CVD progresses.

Toll-like receptors (TLRs) are involved in innate immunity, and a number of isoforms exist from TLR1-10 in humans [47]. The evolutionary purpose of TLRs are to appropriately respond to pathogens by inducing an inflammatory immune response. All of these TLRs can bind to a number of bacterial components [48]: for example, TLR4 to lipopolysaccharides (LPS), a component of the outer membrane of Gram-negative bacteria; TLR2 to lipopeptides, and TLR3 to double-stranded RNA [48]. In macrophages, LPS can trigger an inflammatory response at very low concentrations, such as 100 pg/mL [49], which is a physiologically relevant concentration in human serum following certain nutritional interventions, such as high-fat feeding with animal products [50,51]. TLRs are found ubiquitously across most tissue and cell types, including the cardiovascular system [52,53,54,55,56]. Their role in driving the pathogenesis of CVD is recognized as a critical component of the molecular disease process [57], driving both atherosclerosis and heart failure, particularly TLR2 and TLR4 [58]. While tuned to pathogenic stimuli, a number of exogenous and endogenous ligands which are mediated by diet appear to also lead to their activation or upregulation [59,60]. For example, TLR2 and TLR4 can be upregulated or activated by oxidized low-density lipoproteins (oxLDL) [61,62,63], while angiotensin (Ang) II can mediate TLR4 [64,65], all of which can be regulated by diet (to be discussed). Because of these ligands which TLRs can react with, and due to the inflammatory response that ensues, diet has the capability of modulating TLR-mediated signaling in the cardiovascular system, promoting CVD risk (Figure 2).

The role of the other TLRs in the context of diet and CVD is less clear; however, all TLRs may be upregulated in adipose tissue of obese mice [66], and TLR8 in adipose tissue of diabetic humans [67]. While viral double-stranded RNA is classically considered a TLR3 agonist, RNA released from necrotic cells can also activate TLR3 [68]. Thus, in a state of acute CVD, TLR3 can be activated from neighboring apoptotic cells [69]. TLR9 may be protective in adipose tissue, as its deletion exacerbates the inflammatory effects of obesity [70], while others, such as TLR1 and TLR6 in macrophages may become active, due to dimerization with TLR2 following palmitate stimulation [71], a type of saturated fatty acid. In the context of diet and CVD, TLR2 and TLR4 are the dominant TLRs identified in the literature, and appear to play a predominant role compared with other isoforms, particularly TLR4, which will be the primary focus in this review.

### 2.1. Molecular Signaling of TLR

TLRs can act in a MyD88-dependent pathway (a scaffolding protein just downstream of the receptor) via both TLR2 and TLR4, and a MyD88-independent pathway via TRIF, via TLR4 [47]. In the context of downstream terminal signaling, the differences in these pathways are not of substantial relevance in the context of this review, as similar molecular outcomes occur, these being reactive oxygen species (ROS)-producing NADPH-oxidase (NOX) activation [72,73,74,75] and mitochondria-derived ROS production [76], as well as inflammatory signaling via mitogen-activated protein kinases (MAPKs) and nuclear factor-κB (NF-κB) [74,77,78,79,80,81,82] (Figure 2), proteins found in all cells of the cardiovascular system. While NOX activation may be due to protein–protein interaction with the TLR protein itself [73,75], both MyD88-independent and -dependent pathways converge with TRAF6, a protein which, when ubiquitinated, can activate transforming growth factor-β-activated kinase (TAK)1, a mitogen-activated protein kinase kinase kinase (MAPKKK), involved in the phosphorylation of IκB kinase (IKK) [83], upstream of NF-κB complex, as well as terminal MAPKs: c-Jun N-terminal kinase (JNK) and p38MAPK [84,85]. Crosstalk also occurs between ROS and inflammatory pathways, which can exacerbate TLR signaling. For example, ROS produced by NOXs or mitochondria can cause autophosphorylation of IKK, due to redox modification of reactive cysteine residues [86]. Additionally, ROS can lead to activation of apoptosis signal-regulating kinase 1 (ASK1) [87], a redox-sensitive MAPKKK, upstream of terminal p38MAPK and JNK, leading to their activation [88]. With respect to the terminal MAPK extracellular signal-regulated protein kinase (ERK)1/2, TLR does indeed induce its activation; however, TAK1 and ASK1 are not involved in this signaling, but rather, the MAPKKK, Raf, is a parallel pathway which is involved [88].

Sustained and chronic activation of terminal MAPKs has mostly a pathological role in the cardiovascular system. ERK1/2 is involved in cardiac hypertrophy and vascular smooth muscle cell (VSMC) proliferation in atherosclerotic lesions [89,90,91], while JNK and p38MAPK drive cardiac apoptotic signaling leading to fibrosis [92]. In the context of atherosclerosis, all three MAPKs in macrophages may decrease cholesterol efflux and drive foam cell formation [92,93], and all three MAPKs are pathologically involved in the development of atherosclerotic lesions in the endothelium [90,94,95]. Nuclear transcription factors, the activator protein-1 (AP-1) family (downstream of JNK and p38MAPK), as well as the p65/p50 subunit of NF-κB, act in the nucleus in a cooperative manner to increase inflammatory cytokine and chemokine expression [96,97,98], among other proinflammatory proteins.

### 2.2. Role of TLRs in CVD

The pathological effects of chronic or excessive TLR4 signaling in CVD encompass an interplay between both the cells of the cardiovascular system and immune cells, both of which have increased TLR4 expression and neither of which act independently. In the myocardium, upregulated TLR4 signaling drives fibrosis and cardiomyocyte hypertrophy and apoptosis [76,99,100], which can promote heart failure. In endothelial cells, in the context of atherosclerosis, TLR4 signaling leads to synthesis of the chemokine monocyte chemoattractant protein (MCP)-1 [101] which can attract macrophages, while synthesis of glycoproteins: intracellular adhesion molecule (ICAM)-1, vascular cell adhesion molecule (VCAM)-1 and E-selectin [102,103] facilitates their adhesion and localization in the sub-endothelial space [104]. TLR4 upregulation in the macrophage drives foam cell formation in the sub-endothelial space after phagocytosis of oxLDL [105,106], leading to plaque formation and atherosclerosis. Illustrative of these effects, genetic TLR4 ablation in murine models protects animals from developing both heart failure [107] and atherosclerosis [108]. In high-fat-diet-fed mice, genetic deletion of TLR4 preserved heart function compared to TLR4^+/+^ animals [109]. Phosphorylation of JNK and IKK in the heart was reduced in TLR4^−/−^ animals vs. TLR4^+/+^, as was ROS production. Similar to TLR4, in a number of animal models, genetic TLR2 or MyD88 deletion substantially protects mice from atherosclerotic lesion development, mediated primarily through reduced macrophage recruitment into the endothelium [108,110,111,112,113]. In an investigation by Liu et al. [112] with ApoE^−/−^ mice, this protective effect occurred despite isolated macrophages from both TLR2^−/−^ and TLR2^+/+^ expressing similar propensity for foam cell formation. Inhibition of TLR2 also appears to protect the heart from myocardial infarction [114] and ischemia-reperfusion injury [115]. 

### 2.3. Potential Role of Diet-Derived Endotoxins from Animal Foods in CVD Development

It has been recognized in recent years that Western dietary patterns are associated with increased serum endotoxin (e.g., LPS) concentrations [116]. The consumption of animal products likely contributes to this, as animal products, including minced beef, butter, cream, pork, turkey and ice cream, contain appreciable quantities of LPS [117,118,119,120]. These findings are not unexpected, considering LPS is derived from microbes; therefore, foods which facilitate substantial microbial growth and proliferation can contain considerable endotoxin quantities. LPS appears to withstand boiling at 100 °C for up to 30 min, while after this time its TLR4-stimulating activity and subsequent NF-κB induction tends to decrease [118]. Additionally, in conditions meant to simulate the gastric environment, a low pH of 1 induced by hydrochloric acid treatment as well as protease treatment did not reduce LPS-stimulated TLR4-mediated NF-κB induction. Thus, LPS can persist in these foods, even with typical cooking methods and digestion. Further, there is evidence that LPS concentrations may increase as storage time increases, as demonstrated with raw, unpasteurized milk under cold-storage conditions [121].

In humans, the consumption of toast with 50 g of butter resulted in a transient increase in plasma endotoxin concentration (50% increase), from a median of 8.2 to 12.3 pg/mL [119]. In vitro, 10 pg/mL of LPS, a physiologically relevant concentration, was able to stimulate inflammatory cytokine release in monocytes [119]. When human plasma was spiked with this concentration of LPS, human aortic endothelial cells expressed the leukocyte adhesion molecule, E-selectin [119]. Thus, even very low concentrations of LPS in serum could induce an inflammatory response. Dietary fat modulates LPS concentrations, as postprandial changes in triglycerides as well as chylomicrons tend to parallel changes in endotoxin concentrations [50,122,123], with particular exacerbation in subjects with obesity or an underlying metabolic pathology. This is a noteworthy observation, because absorption of dietary lipids, especially saturated fatty acids [124], participates in the translocation of LPS through the gut wall via chylomicrons [125]. Indeed, as part of a high-fat diet, chicken and pork supplementation appears to increase plasma endotoxin concentrations and hepatic TLR4 expression to a much greater degree than soybean supplementation in mice [126]. Meats higher in fat (e.g., beef) also appear to result in increased systemic inflammation in humans to a greater extent, compared with leaner game meats [127,128]. It is unlikely that these endotoxins are derived from host microbial populations, because intestinal absorption of fat occurs primarily in the jejunum and duodenum [129]. This location is of importance, because this section of the small intestine contains substantially lower concentrations of microbes (10^3^–10^4^ bacteria/mL), which are transient, non-local populations, compared with the ileum (10^8^ bacteria/mL) and the colon (10^10^–10^11^ bacteria/g), which contain more permanent microbial residents [130].

The ingestion of sugar-free cream (300 calories) also resulted in a substantial rise in endotoxins (+45%), which peaked at 3–5 h in healthy human subjects [120]. This rise paralleled increased TLR4 expression in isolated peripheral blood mononuclear cells (PBMCs) and NF-κB activity. It is important to note that 75 g (300 calories) of glucose ingestion also resulted in an inflammatory response, albeit non-TLR4 mediated, likely due to hyperglycemia and potential oxidative stress [131,132,133]. The inflammatory response to glucose also appeared earlier in the postprandial phase (1 h) compared with cream, which had a lag time of ~3 h [120], likely due to the delayed digestion of lipids. Interestingly, an equal calorie consumption of orange juice did not result in an inflammatory response compared with glucose alone, likely due to the protective effects of ascorbic acid or polyphenols. This occurred despite substantial endotoxin content in orange juice (8.5–17 ng/mL) compared with cream (10.4–20.8 ng/mL). Again, the effect of lipid ingestion appears to play a major role in whether or not these endotoxins are absorbed. For example, a high-fat meal containing sausage, eggs and hashbrowns (47% fat, endotoxin load: 420–840 ng/mL) resulted in a 42% increase in plasma endotoxin concentrations compared with a low-fat AHA meal (27% fat), despite a higher endotoxin load in the AHA meal (570–1140 ng/mL) [51]. While the AHA meal contained less saturated fat, it also contained plant foods, such as oatmeal, peanut butter, raisins and orange juice, which may have been mostly responsible for these protective effects.

Illustrative of the protective effects of plants, the consumption of this identical high-fat meal alongside orange juice blunted the rise in plasma endotoxin concentrations, which corresponded with reduced PBMC TLR4 expression compared to water and glucose co-consumption [134]. There was also partially attenuated ROS generation with orange juice consumption compared to both water and glucose co-consumption, with the high-fat meal. A reduction in NOX2 expression as well as reduced phosphorylation of p38MAPK was observed with orange juice consumption in these cells. The explanation for this reduction in plasma endotoxin concentration is not fully clear, but some evidence suggests a reduced LPS bioavailability when plant foods are co-consumed, perhaps due to the phytochemical or fiber content [135,136]. Therefore, high-fat diets which emphasize animal product consumption at the expense of plant-food consumption, such as the increasingly popular “carnivore” diet and the ketogenic diet, as well as the Atkins diet [137,138], may be particularly prone to elevated blood endotoxins and may increase the risk of CVD. Indeed, when subjects with established coronary artery disease switched from a vegetarian diet to an Atkins diet on their own accord, the severity of their disease increased by 52%, compared with a 21.8% improvement in the vegetarian diet group [46].

It should be noted that while much of the aforementioned postprandial data refers to the vasculature, this is due to methodological limitations in assessing the myocardium. However, considering that endotoxins are equally pathological in cardiomyocytes and cardiac tissue [139,140,141], it is expected that these pro-oxidative and inflammatory effects in the postprandial phase likely occur systemically within the cardiovascular system and would also be pathological in the heart. Indeed, serum endotoxins were predictive of atrial fibrillation, and as part of a Mediterranean diet, only fruits and legumes were significantly associated with reduced LPS concentrations, while meat consumption trended (*p* = 0.085) towards being associated with increased LPS concentrations [142]. Additionally, young healthy controls and centenarians both had lower serum endotoxin concentrations compared with individuals that had a myocardial infarction [143]. In isolated human cardiac tissue, treatment with LPS significantly reduced contractility and increased inducible nitric oxide synthase (iNOS) [144], a pathological contributor of superoxide (O_2_^−^)-induced oxidative stress [145,146,147].

### 2.4. Oxidized LDL from Diet: TLR-Mediated Effects

Cholesterol oxides are similar to cholesterol in structure; however, they are modified, due to oxidative reactions, to contain hydroxyl and epoxide groups, for example [148]. Cholesterol oxides can be consumed in the human diet and incorporated into chylomicrons, and assimilate into LDL particles in human serum [149]. These oxides are found in a variety of animal products, including beef, turkey, butter and eggs [148,150]. Cholesterol oxide concentrations in these foods increase in a time-dependent manner during storage, and are also increased following cooking. For example, oven-cooked beef, veal and pork resulted in an increase in cholesterol oxide content by 352%, 540%, 421%, respectively [151]. In addition to dietary cholesterol oxides, extracellular ROS derived from cells can oxidize endogenously produced cholesterol [152,153]. Cholesterol oxidation susceptibility ex vivo was found to be increased 37–39% following the consumption of egg yolks in humans, for example [154,155]. In a similar manner, patients with CVD also have a higher susceptibility for cholesterol to oxidize, compared to healthy controls [156]. Thus, under inflammatory conditions which coincide with increased oxidative stress, it could be expected that LDL particles would be oxidized in vivo, increasing the circulating pool of oxLDL and exacerbating the inflammatory response.

In addition, having higher serum cholesterol concentrations also results in increased oxLDL, due to the intrinsic fact that there is simply more LDL available to be oxidized. For example, oxLDL concentrations increase in a stepwise manner in human subjects with borderline-elevated serum LDL and high serum LDL compared to healthy controls [157]. Endothelial-dependent vasodilation was diminished by 20% in these human subjects with elevated LDL concentrations, likely due to oxLDL concentrations, since native LDL does not appear to diminish endothelial nitric oxide synthase (eNOS) activity, an enzyme involved in mediating vasodilation via release of nitric oxide (NO), while oxLDL did diminish eNOS activity [158]. Thus, consumption of animal products can (1) introduce dietary cholesterol oxides, (2) increase the propensity of cholesterol to oxidize, and (3) increase serum LDL, creating a greater opportunity for LDL oxidation.

In animal models, the consumption of cholesterol oxides as part of a high-cholesterol diet increased fatty streak lesions in the aorta of rabbits by 100% [159], 32% in LDLR-deficient mice, and 38% in ApoE^−/−^ mice [160]. These detrimental effects may be due to TLR activation, as oxLDL particles mimic microbial pathogen-associated molecular patterns which can be recognized by TLRs [161], particularly TLR4 [62]. In Wistar rats, the consumption of oxidized cholesterol for 14 weeks resulted in a ~10% increase in left ventricle infarct size compared to control animals, while heart failure induction via isoproterenol was exacerbated by oxLDL (56% infarct size) compared with a standard diet without oxLDL (36% infarct size) [162]. These effects were tied to increased myocardial TLR4 mRNA, which mirrored changes in infarct sizes. Indeed, compared with other CVD risk factors, including serum lipoproteins alone, oxLDL concentration is a greater predictor of CVD events, even in otherwise healthy individuals [163].

## 3. Saturated Fat from Animal Foods: Molecular Consequences beyond Increased LDL Cholesterol

Palmitate is a major saturated fatty acid found in oils, particularly saturated fat-rich oils, but it is also found in significant quantities in dairy products (e.g., butter and cheese), eggs and some meats [164]. This is of significance, since saturated fats in the American diet are primarily derived from cheese, beef, other fats and oils, milk, and luncheon/sausage/other processed meats [165]. Thus, palmitate is a major saturated fatty acid in the American diet. In vitro, cardiomyocytes, endothelial cells and VSMCs all experience deleterious inflammatory effects following palmitate treatment [166,167,168]. 

Excessive saturated fat consumption results in increased fasting free fatty acid (FFA) serum concentrations, especially palmitate, due to reduced peroxisome proliferator-activated receptor (PPARs) α and γ activity, resulting in reduced fatty acid oxidation and reduced storage in adipose tissue, respectively, due to preference of these PPARs for monounsaturated fats [169]. Additionally, saturated fat consumption results in poorer triglyceride assimilation, due to the preference of diacylglycerol acyltransferase, an enzyme involved in triglyceride formation, for monounsaturated fats [169]. Thus, an elevation of FFAs is expected in subjects that consume saturated-fat-rich diets. Indeed, this is observed, as a four-week isocaloric, weight-maintaining ketogenic diet (15% protein, 5% carbohydrate, 80% fat) resulted in significantly greater FFA concentrations compared with four-week consumption of a minimally processed baseline diet (15% protein, 50% carbohydrate, 35% fat) [45], or two weeks of an isocaloric, weight-maintaining plant-based diet (~14% protein, ~75% carbohydrate, ~11% fat) compared to a ketogenic, animal-based diet (~16% protein, ~10% carbohydrates, ~74% fat) [170]. In the four-week intervention [45], serum C-reactive protein (CRP) increased on the ketogenic diet, while in the two-week intervention [170], CRP did not change on the ketogenic diet from baseline, but the plant-based diet resulted in significantly lower CRP concentrations in comparison. A ketogenic-type diet resulted in deleterious cardiac effects in spontaneously hypertensive rats [171], ischemia-reperfusion injury [172,173], diabetes [174,175], long-term ketogenic-diet feeding [176], and two-week ketogenic feeding [177]. Interestingly, protection was observed in a transaortic constriction (pressure-overload) model [178,179].

The detrimental effects of saturated FFAs are unlikely due to receptor–ligand interaction of TLRs; however, TLR2 and TLR4 do seem to play a role, albeit not as direct receptors. It has been demonstrated that palmitate is not a true TLR4 ligand [180,181], but rather, that it exacerbates the TLR-mediated inflammatory response [180,182]. Lancaster et al. [180] convincingly demonstrated that in TLR4^−/−^ macrophages, palmitate treatment was not solely responsible for inducing an inflammatory response. However, when cells were pretreated with TLR2 and TLR3 agonists, [47], only then was palmitate able to induce an inflammatory response due to this priming. In the much more complex environment in vivo, this priming is expected to occur, since dietary components and other endogenous products may act on TLRs. Nonetheless, the authors hypothesized that the initial inflammatory response with TLR4^+/+^ macrophages and palmitate in vitro was due to low endotoxin contamination in BSA (used to conjugate palmitate) which caused this initial priming.

In contrast, Lee et al. [183] demonstrated that the saturated fatty acid sodium laurate, which is water soluble and does not require BSA solubilization, activated TLR2 and TLR4 in macrophages. Huang et al. [71] also showed that very-low-dose BSA (0.25%) was insufficient to elicit an inflammatory response in macrophages; however, inflammation increased with palmitate. Mo et al. [184] showed in humans that after the consumption of a high-fat meal, plasma endotoxin concentrations were not detected. However, increased inflammatory cytokines were detected which were exacerbated by lipoprotein lipase treatment of whole blood, liberating FFAs from triglycerides, resulting in a substantially greater inflammatory response. It was noted that serum palmitate concentrations rose at hour 3 compared to baseline and peaked at hour 6, indicating that this lipotoxic response is of relevance and may indeed activate TLR4. Indeed, infusion of palmitate in TLR4-blunted mice demonstrated substantially reduced myocardial injury, inflammatory cytokine protein expression, and cardiac fibrosis, compared to wild-type animals with intact TLR4 gene [167]. Authors note that palmitate displayed binding affinity towards the MD2 subunit of TLR4, leading to reaction with MD2 and TLR4 activation.

### 3.1. Lipotoxicity

**Figure 3:**
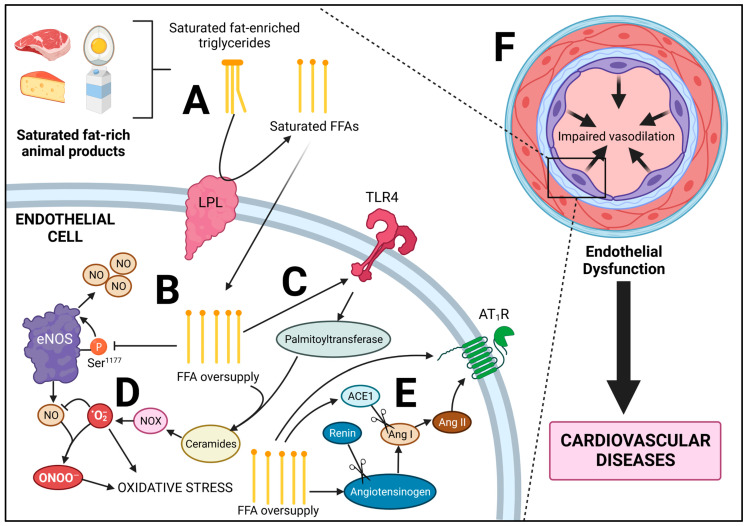
Endothelial lipotoxicity mediated by consumption of saturated-fat-rich animal foods. (**A**) Excessive consumption of saturated fats from animal foods leads to synthesis of triglycerides which are enriched with saturated fatty acids. Lipoprotein lipase (LPL) in endothelial cells liberates fatty acids from triglycerides, resulting in free fatty acid (FFA)-uptake by the cell. (**B**) Excessive FFAs in the cell results in FFA oversupply and resulting lipotoxicity. This can result in reduced phosphorylation of endothelial nitric oxide synthase (eNOS) at Ser^1177^, due to modulation of upstream regulators of Akt. (**C**) FFA oversupply can exacerbate primed TLR4-mediated inflammatory- and oxidative-stress signaling. TLR4 activation increases the activity of serine palmitoyltransferase, leading to increased ceramide synthesis. (**D**) Increased intracellular ceramide accumulation can result in increased NADPH-oxidase (NOX) activity, resulting in increased superoxide (O_2_^−^) production and a reduction in NO bioavailability, due to peroxynitrite formation (ONOO^−^). These reactive oxygen and reactive nitrogen species increase cellular oxidative stress. (**E**) FFA oversupply increases the renin angiotensin system (RAS) via increased angiotensinogen, angiotensin-converting enzyme (ACE)1 and angiotensin II type-1 receptor (AT_1_R) signaling. (**F**) Cumulatively, the effects of downregulated eNOS, reduced NO, exacerbation of inflammatory stimuli, increased oxidative stress and increased AT_1_R signaling which exacerbates these effects, results in endothelial dysfunction and impaired vasodilation, a hallmark of cardiovascular disease and indicator of diminished vascular health.

Because animal-based diets such as the Atkins, ketogenic, or carnivore diet derive calories from animals and less so from plants, carbohydrate quantity is inherently low, while fat content, particularly saturated fat content, is high. Because of this, high-fat diets are associated with elevated fasting FFA concentrations (~0.8 mmol/L) in otherwise healthy individuals, for reasons discussed in the preceding section, which is higher than that of insulin-resistant lean individuals (~0.6 mmol/L), and comparable to obese (0.6–0.8 mmol/L) and diabetic (0.7–0.9 mmol/L) subjects [185]. Several clinical investigations demonstrate this increase in plasma FFAs following animal-product-based diets [45,186,187], which ties with increased intramyocellular lipid accumulation [188], the main driver of insulin resistance [189]. Saturated fat is much more lipotoxic than unsaturated fats, due to preferentially increased intracellular fatty acid deposition via upregulated diacylglycerol synthesis, as well as increased ceramide synthesis, a fatty acid metabolite [169]. Indeed, individuals consuming saturated-fat-rich diets tend to be more insulin resistant following a carbohydrate challenge [45,190,191], which falls in line with several animal studies [192,193,194,195]. Lipid infusion in itself can result in insulin resistance in humans, which mimics these effects [196,197]. It has also been documented that high-protein, but not high-fat diets, can increase de novo lipogenesis of palmitate in the liver [198]. With regard to the cardiovascular system, animal-food-based diets tend to diminish endothelial function [44]. Indeed, healthy subjects that underwent 4 h lipid infusion or a 5-day animal-food-based diet (the Atkins diet) experienced aortic stiffness to the same degree as those afflicted with obesity [199]. Thus, elevated FFAs play a critical pathological role in the endothelium, and a number of mechanisms can drive this effect (Figure 3).

### 3.2. Lipotoxicity of the Endothelium

The enzyme eNOS, produces NO, a potent vasodilator [200]. There are numerous regulatory mechanisms which mediate eNOS activity, including phosphorylation sites, growth factors, protein–protein interactions, shear stress and ROS [200,201,202]. Under lipotoxic conditions, eNOS activity is impaired and NO bioavailability is significantly reduced [203,204]. NO bioavailability can be considered a proxy for endothelial health. Indeed, a reduction of NO drives hypertension, due to increased vasoconstriction [205], and reduced NO is an indicator of pro-atherogenic conditions [206].

In human endothelial cells, insulin signaling results in Akt phosphorylation, leading to eNOS phosphorylation at Ser^1177^, increasing eNOS activity [207]. However, in healthy individuals infused with FFAs, significant reduction in plasma NO was observed following both short-term (2–4 h) and long-term (8 h) infusion after insulin was infused to stimulate eNOS [203]. Additionally, NO synthesis was also impaired in bovine aortic endothelial cells pretreated with or without 100 μmol/L FFA for 3 h followed by eNOS stimulation with 100 nM insulin [204]. Five minutes following the addition of insulin, Akt phosphorylation and subsequent eNOS phosphorylation at Ser^1177^ were blunted by FFAs. These effects were due to increased IKK phosphorylation, an upstream regulator of NF-κB signaling. eNOS is typically in protein–protein interaction with heat shock protein-90 (Hsp-90); however, increased IKK activity disrupts this interaction, driving the reduction in eNOS activity [208]. In addition to this protein–protein interaction, in spontaneously hypertensive rats, a ketogenic diet significantly increased blood pressure and reduced eNOS expression in mesenteric arteries [209]. These effects were attenuated with NF-κB inhibition, a regulator of tumor necrosis factor (TNF-α) which can transcriptionally downregulate total eNOS protein expression [210].

The detrimental effects of FFAs may be in part due to ceramide synthesis, an intracellular metabolite of fatty acid metabolism in the cell, which can accumulate under lipotoxic conditions of FFA oversupply [211], particularly saturated FFAs such as palmitate [212,213]. Infusion of lard oil (SFA-rich), but not soybean oil (SFA-poor), increased ceramide synthesis in skeletal muscle in a TLR4-dependant manner [214]. Indeed, TLR4 activation increases ceramide synthesis via increased serine palmitoyltransferase expression [215,216], the first rate-limiting enzyme in the synthesis of ceramides [217]. Ceramides in small coronary arteries increase the production of O_2_^−^ mediated by NOX [218], which can bind to NO, forming the radical ONOO^−^ [219]. This led to endothelial dysfunction in these coronary arteries [218]. Interestingly, ceramides appear to increase eNOS expression at the transcriptional level; however, this compensation in eNOS expression was insufficient in overcoming O_2_^−^-induced reduction in NO bioavailability [220].

### 3.3. Consumption of Animal Foods and Saturated Fat: A Link to the Renin-Angiotensin System

The renin–angiotensin system (RAS) is a major target in cardiovascular therapeutics, particularly with angiotensin-converting enzyme (ACE)1 inhibitors and Ang II type-1 receptor (AT_1_R) blockers [221]. Ang II is a primary product of RAS, and its pathological effects via AT_1_R are well known [222,223], with additional evidence for its role in also mediating TLR4 signaling [224]. RAS components include angiotensinogen, which is cleaved by renin to form Ang I, which is cleaved further by ACE1 to form Ang II which can then act on AT_1_R [225]. Ang II can be further cleaved by ACE2 to form Ang (1–7), which is protective via its action through the Mas receptor. All components of RAS are found in most tissues of the human body, including the entire cardiovascular system [226]. In the cardiovascular system, AT_1_R tends to increase oxidative stress via increasing NOX- and mitochondrial-derived ROS, as well as inflammation in all cell types [227]. AT_1_R can reduce NO derived from eNOS in endothelial cells via (1) reducing NO bioavailability due to increased ROS, (2) reducing eNOS phosphorylation at Ser^1177^, and (3) protein–protein interactions with AT_1_R and eNOS [228,229]. AT_1_R signaling in VSMCs can independently promote vasoconstriction [230], in addition to AT_1_R-mediated VSMC hypertrophy [231] and migration [232], all of which appear to be mediated by oxidative stress, pathologically impacting vascular function. AT_1_R signaling in cardiomyocytes of the heart increases hypertrophy and pathological remodeling [233,234], potentially leading to dysfunction. Hence the use of AT_1_R blockers in patients with heart failure [222].

As previously discussed, low-carbohydrate diets tend to be rich in saturated fat, driving increased serum LDL cholesterol concentrations [235] and fasting FFAs [185]. However, a rise in LDL cholesterol can pathologically increase AT_1_R in vivo and in vitro [236,237,238]. AT_1_R can also be increased by oxLDL, as observed in endothelial cells, in which AT_1_R was transcriptionally mediated by NF-κB [239]. Caffeic acid phenethyl ester, an NF-κB inhibitor, prevented oxLDL-mediated AT_1_R transcription in this model. In humans, 6-week consumption of a high-fat diet (45% fat) rich in saturated-fat-containing red meat, sausage, bacon, and full-fat dairy products resulted in an increase in serum LDL, ACE1 and an increase in ACE1 mRNA in adipose tissue [240]. In murine immortalized adipocytes, palmitate treatment increased angiotensinogen and AT_1_R mRNA expression, which also increased Ang II secretion [241]. The role of AT_1_R in mediating the pathological effects of FFA is made clear by Watanabe et al. [242] in which healthy human subjects received lipid infusion with or without losartan (AT_1_R antagonist) or perindopril (ACE1 antagonist). Endothelial-dependent vasodilation with acetylcholine was impaired by the lipid infusion; however, both losartan and perindopril abolished this effect, indicating that both Ang II synthesis and Ang II-AT_1_R binding are increased when serum FFAs are increased. Thus, a diet containing saturated-fat-rich animal products could drive several pathological pathways in the cardiovascular system, including lipotoxicity and increased RAS.

## 4. Animal Products and the CVD-Promoting Trimethylamine-N-Oxide Molecule

Both choline and carnitine are derived from endogenous synthesis as well as dietary intake; however, choline dietary intake is required to meet physiological needs [243,244]. These nutrients have important physiological roles; carnitine, for example, facilitates fatty acid metabolism via transport into the mitochondria [245], while choline has an array of functions, including acetylcholine synthesis and cell membrane synthesis [246,247]. Choline and carnitine are predominantly found in animal-based foods, such as beef, eggs and dairy [243,244]. With respect to choline, however, sufficient quantities can be obtained from plant-based foods if well planned, including from soy, potatoes, beans and grains [243] In excess, both choline and carnitine, once metabolized by host gut microbiota, result in the synthesis of trimethylamine [248]. The liver further metabolizes this product to trimethylamine-N-oxide (TMAO), a compound tightly associated with atherosclerosis [249]. The consumption of red meat resulted in the synthesis of TMAO in omnivorous subjects, while vegan subjects did not experience this increase due to differing microbial populations preventing trimethylamine synthesis [248]. In ApoE^−/−^ mice, carnitine ingestion significantly increased aortic plaque formation compared to animals which did not consume carnitine [248]. The pro-atherogenic effects of TMAO appear to be mediated by increased cellular oxidative stress as well as MAPK and NF-κB signaling, driving an inflammatory response in endothelial cells, VSMCs, and macrophages [249].

For example, in both human aortic endothelial cells and human VSMCs, TMAO treatment in vitro significantly increased MAPK and NF-κB activation [250]. Indeed, the adhesion of leukocytes was found to be increased with TMAO treatment following co-culture of both endothelial cells and leukocytes together. Macrophages, for example, experience a phenotypic switch towards a more inflammatory phenotype following TMAO exposure [251], which may even increase TLR4 expression [252]. With respect to VSMCs in vitro, TMAO treatment dose-dependently increased calcification of cells, due to phenotypic changes towards osteoblast-like cells, which was abrogated by the inhibition of NF-κB [253]. In isolated rat aortic rings ex vivo, TMAO similarly increased aortic calcification in a dose-dependent manner which paralleled in vivo formation of vascular calcification in rats with chronic kidney disease that underwent TMAO injection [253]. In support of this data, in humans with chronic kidney disease, serum TMAO concentrations were closely tied to aortic arch calcification [253]. TMAO concentrations in humans are also closely tied to heart failure severity and mortality [254]. Animal models demonstrate that increased TMAO can drive left ventricular hypertrophy and fibrosis, cardiac inflammation and oxidative stress, and exacerbate mitochondrial dysfunction [255]. Indeed, cardiomyocytes that underwent TMAO treatment had impaired contractility, due to poor calcium handling, in addition to increased oxidative stress [256]. These effects may be attributed to altered energy dynamics, due to reduced mitochondrial capacity for β-oxidation [257] coupled with impaired glycogen utilization [256]. Thus, TMAO is of significant pathophysiological and clinical relevance in CVD.

Eggs and red meat appear more closely associated with TMAO concentrations in white Americans, while in Asians, fish and shellfish had greater associations with circulating TMAO [258]. In an interventional study, the consumption of red meat significantly increased urinary TMAO concentrations after four weeks compared to white meat and non-meat protein source interventions [259]. Interestingly, a carnitine challenge increased TMAO in both the red meat and white meat interventional groups, but not the non-meat protein-source group. This suggests that while white meat may not increase TMAO directly, gut microbiota populations in these individuals were still able to synthesize TMAO. Thus, following the consumption of carnitine/choline-rich foods (e.g., eggs, red meat, dairy), TMAO would still be produced in a predominantly white-meat-containing diet. In contrast, the non-meat-containing diet appeared to have a favorable gut microbiota profile which did not produce TMAO. Overall, plant-based diets tend to be associated with reduced TMAO concentrations, while the inverse is true with respect to animal-based diets [260]. Illustrative of this finding, an eight-week vegan diet significantly reduced plasma TMAO within one week in obese subjects, levels which rebounded to baseline levels at week 12, following a return to their normal diets [261]. In a randomized cross-over investigation, an animal-food-rich Atkins diet significantly increased TMAO concentrations compared to a plant-based Ornish diet [262]. In a postprandial study, the consumption of eggs, beef and fish all significantly increased postprandial TMAO concentrations compared to a fruit meal [263].

## 5. Considerations for Fish Consumption

Fish intake overall has been associated with reduced mortality as well as reduced CVD incidence [264,265]. However, it is interesting to note that population studies reflect that in the United States, there is a U-shaped mortality curve, with 20 g/d of fish appearing optimal, while increasing intake appears to increase mortality [266]. This trend did not occur in Japanese populations, as intake appeared more linearly associated with reduced CVD mortality. This suggests possible differences in preparation method. Fish tends to be lower in saturated fat and provide Omega-3 fatty acids, which have independent protective effects. However, fish is also a source of carnitine and choline and has higher concentrations than plant-based foods [243,244]. Indeed, fish consumption postprandially increased TMAO concentrations 46–62 times higher than the consumption of fruits, eggs or beef [263]. Evidence overall suggests that a plant-based diet may have higher therapeutic potential than a pescatarian diet in reducing CVD risk, possibly due to the detriments of TMAO.

For example, in the Adventist Health Study-2, vegans were the only dietary group which had a normal body weight (23.6 BMI) compared to pesco-vegetarians, who were, on average, overweight (26.3 BMI) [267]. Type 2 diabetes prevalence was also 65% greater in pesco-vegetarians compared to vegans. Nonetheless, pesco-vegetarians had a lower BMI and type 2 diabetes prevalence compared to non-vegetarians. Hypertension prevalence was 63% lower in vegan non-blacks compared to omnivores, while pesco-vegetarians and semi-vegetarians collectively had an 8% reduced prevalence compared to omnivores [268]. In black vegetarians, a 44% reduction in hypertension prevalence was observed compared to a 6% reduction in pesco-vegetarians.

Sex differences likely exist as well. For example, in the Adventist Health Study-2, ischemic heart disease and overall CVD-related mortality risk was reduced by 55% and 42%, respectively, in vegan males, compared to a 23% and 34% reduction, respectively, in male pesco-vegetarians [269]. However, in females in this cohort, a vegan diet did not confer benefit with respect to ischemic heart disease and overall CVD incidence, while a pesco-vegetarian diet did. Clinical trials are needed to understand these sex differences and the sex response to differing diets. Nonetheless, fish is also a major source of dietary pollutants including lead, mercury and arsenic, which increase blood concentrations of these metals in those consuming 1 kg of fish per week for 26 weeks [270]. Thus, the consequences of these increased heavy metals in pesco-vegetarians may extend beyond CVD risk. While theevidence suggests that a fully plant-based diet may be more efficacious in reducing CVD development compared to a fish-containing diet, clinical studies are needed to determine the comparative efficacy of a pesco-vegetarian diet and a minimally processed, fully plant-based diet.

## 6. Implications and Perspectives

**Figure 4:**
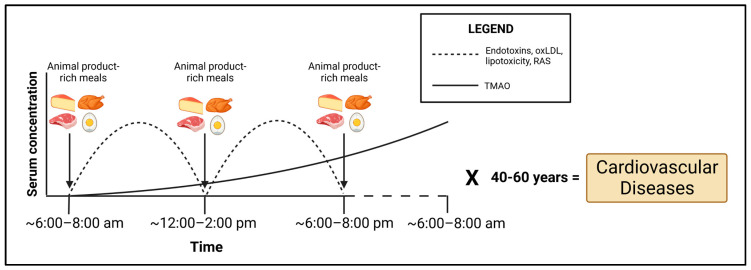
Hypothesized model by which cardiovascular disease is potentially promoted over a lifespan due to frequent postprandial insult caused by consumption of animal products, and the resulting metabolites in serum which mediate the CVD-promoting effects.

Humans in Western societies usually spend a substantial portion of their waking hours in the postprandial phase (<6 h after a meal), whereas fasting (>6 h after a meal) usually occurs during sleep. Most of the pathological consequences of a poor diet tend to occur in this postprandial phase, in which we would expect an increase in endotoxins [51], oxLDL [149], saturated-fat-rich triglycerides [271] and resulting RAS activation (Figure 4). TMAO would be expected to be delayed postprandially, due to the dependence upon microbial metabolism, and indeed, TMAO blood concentrations peak at 24 h postprandially [248], although Cho et al. [263] found TMAO to be increased postprandially in plasma in as little as 2 h. Nonetheless, chronic consumption of choline- and carnitine-rich animal foods would be expected to result in chronically elevated TMAO whether fasting or postprandial, due to this lag time (Figure 4). With respect to palmitate and total FFAs, while these decline immediately postprandially [272], this is due to cellular uptake as well as some triglyceride formation. However, most triglycerides postprandially are derived from the fat of the meal itself and not endogenous levels [273]. Based on typical Western eating patterns, triglycerides would be expected to be abnormally elevated (>177 mg/dL) for 12–14 h a day [274]. Importantly, lipoprotein lipase concentrations steadily rise postprandially following a high-fat meal, peaking at 6 h [273]. The implication of this is that cells of the cardiovascular system are directly exposed to FFAs released from triglycerides, driving lipotoxicity in this postprandial phase if an animal-rich meal is consumed, particularly one rich in saturated fat. The cumulative effects of these pathological dietary elements in the postprandial/fasting state would suggest that over decades of the human lifespan consuming a diet rich in animal-based foods, the sustained insult to the cells of the cardiovascular system induced by these dietary elements would drive the development of CVD (Figure 4).

Coronary fatty streaks can already be identified in adolescent youth [275,276], and cholesterol concentrations were closely tied to lesion formation in individuals whose mean age was only 18 years [277] and in individuals ranging from 6 to 30 years of age [278]. Inflammatory biomarkers were also closely tied to atherosclerotic lesion severity in individuals 25–34 years of age [279]. Thus, CVD clearly develops in childhood adolescence and early adulthood, despite the symptoms of its appearance presenting later in adulthood. However, these pathological perturbations are not necessarily permanent fixtures of our cardiovascular system once present. Thus far, a plant-based diet appears to be the only dietary intervention which could be identified in the literature as able to reverse these pathological changes, regressing atherosclerotic plaques and improving myocardial blood flow [30,31,46,280,281]. While clinical investigations are limited, with more studies of increased rigor and scale urgently needed, the profound clinical outcomes observed in these trials combined with compelling epidemiological data strongly indicate that consuming a predominantly plant-based diet should be a primary clinical strategy in CVD prevention and treatment, as echoed by the AHA [38]. A well-planned vegetarian or vegan diet is nutritionally adequate and healthy, according to the Academy of Nutrition and Dietetics (AND), the organizational body in the United States which provides accreditation to registered dietitians [282]. In a 2016 report released by AND, they highlight that a well-planned, vegetarian or vegan diet is appropriate for all stages of the lifecycle including childhood, lactation, pregnancy, and the remaining stages of life [282]. Thus, in order to appropriately address the CVD epidemic which currently kills ~700,000 people a year annually in the United States [283], steps should be taken over the course of a lifetime, no matter what life stage, to consume a dietary pattern which maximizes the consumption of unprocessed plant-based foods, and minimizes the consumption of animal-based foods.

## 7. Conclusions

Dietary reliance on animal-based foods tends to increase numerous pathological molecular drivers of CVD. These include: (1) activation of TLR4 due to increased serum endotoxins and oxLDL, (2) lipotoxicity, due to increased serum FFAs and intracellular ceramides, (3) upregulation of components of RAS and consequently AT_1_R signaling, and (4) microbial production of TMAO from choline and carnitine caused by unfavorable microbiome profiles due to animal food consumption. The consequences of these events increase inflammatory signaling via MAPKs and NF-κB, while also simultaneously driving oxidative stress due to increased NOX- and mitochondrial-derived ROS. Collectively, these molecular consequences in endothelial cells, VSMCs, cardiomyocytes and macrophages drive CVD. Thus, caution should be taken in consuming diets rich in animal-foods, as this may drive CVD development. Particular concern should be taken with the Atkins, ketogenic or carnivore diets, which maximize the consumption of animal-based foods at the detriment of plant foods. Evidence strongly suggests that the consumption of a plant-based diet will favorably impact CVD risk, and this should remain the predominant public health message. Further clinical research is needed to elucidate other possible mechanisms by which animal products could promote CVDs.

## Data Availability

Not applicable.
